# Stabilizing Active Aluminum (Al^3+^) in Acidic Soils via Biochar-Induced Microbial Niches: Focusing on Denitrifier-Mediated Mechanisms, Efficiency, and Environmental Outcomes

**DOI:** 10.3390/toxics14020157

**Published:** 2026-02-06

**Authors:** Chao He, Tuo Zhang, Shiming Su, Yang Zhang, Xibai Zeng, Yao Qiu, Yaxiong Wen, Shiyong Tan

**Affiliations:** 1 Key Laboratory of Agricultural Environment, Institute of Environment and Sustainable Development in Agriculture, Chinese Academy of Agriculture Sciences, Beijing 100081, China; hechao03@caas.cn (C.H.);; 2 School of Environmental and Life Science, Nanning Normal University, Nanning 530001, China; 3 Hunan Tevos Ecological Technology Co., Ltd., Changsha 410100, Chinashiy-tan@hotmail.com (S.T.); 4 Yuelushan Laboratory, Changsha 410100, China

**Keywords:** acidic red soil, rice husk/sawdust, biochar, active aluminum

## Abstract

The pervasive toxicity of active aluminum (Al^3+^) in acidic red soils threatens agroecosystem sustainability, with conventional chemical stabilizers facing cost and secondary pollution constraints. This study evaluated rice husk/sawdust and their pyrolysis-derived biochar as stabilizers, focusing on microbial synergy. Results showed 3% rice husk biochar (RB) achieved 22.1 ± 1.1% stabilization efficiency within 180 days, outperforming sawdust biochar (12.1 ± 0.8%) and raw biomass. Biochar’s alkalinity and porosity created neutral niches, enriching denitrifiers (*Thiobacillus*, *Arthrobacter*, *Thermomonas*) that elevated pH, promoted Al(OH)_3_ precipitation, and enhanced oxygen-containing functional groups. This work valorizes agricultural waste for long-term Al^3+^ toxicity mitigation.

## 1. Introduction

Widely found in humid subtropical regions, acidic red soil is a key soil type supporting the dryland crops production. However, long-term acid deposition and high-intensity fertilization accelerate soil acidification, which significantly increases soil active aluminum (mainly as Al^3+^) [1,2]. It rapidly acts on the root tip, where it induces cell wall hardening, transmembrane ion homeostasis imbalance, and oxidative stress, inhibiting root elongation and branching, ultimately leading to limited water and nutrient absorption and reduced yield [3,4].

Traditional chemical remediation strategies, exemplified by lime amendments, effectively elevate soil pH to induce aluminum hydroxide (Al(OH)_3_) precipitation, thereby reducing dissolved Al^3+^ activity. However, a significant drawback associated with this approach is the potential induction of soil compaction, which can detrimentally impact soil structure and permeability [5,6]. For the in situ management of aluminum bioavailability risks, a range of soil conditioners offers alternative mechanisms. These include silica-based amendments (enhancing pH buffering capacity); phosphorus-based amendments (facilitating Al^3+^ complexation or precipitation, e.g., as aluminum phosphates); and carbonaceous amendments (e.g., biochar, capable of surface complexation, precipitation, and pH modulation) [7,8]. Collectively, these amendments function by mitigating aluminum bioavailability through enhanced buffering capacity, direct complexation/precipitation of Al^3+^, and alteration of aluminum speciation. Concurrently, microbial remediation presents a promising complementary or alternative strategy for controlling aluminum activity and mobility. Notably, research has demonstrated that aluminum-tolerant plant growth-promoting rhizobacteria (PGPR) can efficiently immobilize soluble Al^3+^. This process is primarily mediated through the complexation and stabilization capabilities of extracellular polymeric substances (EPS) secreted by these bacteria [9].

Solid biomass and biochar, as green stabilization materials, offer the dual benefits of carbon preservation and the in situ stabilization of active aluminum in the red acid soil [10]. Solid biomass such as straw and sawdust contains abundant functional groups to complex with Al^3+^, promoting partial stabilization [11]. Meanwhile, it provides indigenous microorganisms with slow-releasing soluble organic matter, enhancing the secretion of EPS [12]. Biochar (BC), produced through biomass pyrolysis, is extensively employed for heavy metal immobilization in contaminated soils [13]. Critically, mesophilic pyrolysis (350–550 °C) generates biochar with high-density alkaline microporous structures. These pores not only neutralize soil acidity but also establish a pH-buffered microenvironment that supports microbial colonization in highly acidic red soils [14,15]. Notably, existing studies demonstrate that both raw biomass and its derived biochar facilitate long-term Al^3+^ stabilization [16]. Although biochar-based amendments have been widely studied, comparative assessments of raw biomass versus biochar in stabilizing active aluminum, particularly in red acidic soils, remain limited. In addition, questions regarding which functional microorganisms dominate the microdomains on the surface/pores of solid biomass and biochar, and their synergistic stabilization mechanisms with carbon materials, still require systematic exploration.

This study systematically investigated the potential of rice husks (RH), sawdust (SD), and their derived biochars, which were rice husk biochar (RB) and sawdust biochar (SB) produced at 550 °C, as carbonaceous amendments for stabilizing active aluminum in acidic red soil. The selection of RH and SD as candidates was grounded in their dual significance as (i) abundant agricultural wastes in global rice/wood processing regions, and (ii) structurally contrasting matrices for mechanistic comparison. The morphology and physicochemical properties of each material, together with their capacity to stabilize active aluminum, were evaluated via batch experiments. We further resolved the stabilized forms of active aluminum and quantified the effects of amendment dosage and the soil microbial community on stabilization efficacy. The objectives were to identify the optimal carbonaceous amendments for mitigating aluminum toxicity, determine which functional microbial groups enhance Al^3+^ stabilization, and elucidate the synergistic mechanisms between these microbes and the optimal amendments. The findings provide both mechanistic insight and an engineering basis for the development of green stabilization technologies for acidic red soils.

## 2. Materials and Methods

**The process of preparing biomass and biochar.** Rice husks (RH) and sawdust (SD) were obtained from a local farm and a wood-processing facility, respectively. The biomass was passed through a 20-mesh nylon sieve to remove twigs, leaves, and other debris, then repeatedly rinsed with ultra-pure water (UPW; >18.2 MΩ·cm resistivity, purified via reverse osmosis + ion exchange, distinct from distilled water) to eliminate entrained impurities, followed by air-drying at room temperature (25 ± 1 °C) for 72 h. RH and SD were pyrolyzed at 500 °C to produce rice husk biochar (RB) and sawdust biochar (SB). The pyrolysis protocol comprised a process of ramping to 500 °C at 10 °C/min, 120 min isothermal hold, and natural cooling to <80 °C [17]. These materials were subsequently used for the bath experiments of active aluminum stabilization in acidic red soil.

**Experimental Procedures.** Acidic red soil (pH 4.2 ± 0.3) was collected from the surface layer (0–20 cm) of a representative dryland agricultural site in southern China (Hunan Province; longitude: 113°00′ E, latitude: 28°12′ N), as well as from the location of the experiment. Prior to the experiment, the soil was air-dried, homogenized, and sieved to <2 mm. For the batch experiment, 100.0 g of processed soil (oven-dry basis) was mixed with RH, SD, RB, or SB at 1%, 2%, or 3% (*w*/*w*) in 1 L open glass jars. Soil incubation was conducted from June to December 2024 (180 days total) under controlled conditions (25 ± 1 °C, 60% water-holding capacity). Control groups comprised inactivated treatments of RH, RB, SD, and SB at 1%, 2%, and 3% (*w*/*w*) application rates. All treatments were prepared in triplicate (*n* = 3). Subsamples were collected at predetermined intervals (days 0, 30, 60, 90, 120, and 180).

**Physico-chemical analysis.** Soil samples were collected and oven-dried at 105 °C for 48 h prior to routine physicochemical characterization. Exchangeable acidity and H^+^ were determined as described [10,18]; active aluminum extraction followed [10], with quantification by ICP-OES (Agilent 5110 VDV, Agilent Technologies, Santa Clara, CA, USA) [19]. In short, exchangeable acidity (cmol/kg) was determined by equilibrating 10.0 g of air-dried soil (<2 mm) with 100 mL of 1.0 mol/L KCl for 1 h; the filtrate was titrated with 0.01 mol/L NaOH to a phenolphthalein endpoint (pH 8.3) under N_2_ to minimize CO_2_ interference. Exchangeable H^+^ (cmol/kg) was measured by adding 1.5% NaF to 50 mL of the KCl extract to complex Al^3+^ and liberate Al-bound H^+^, followed by NaOH titration to pH 8.3. Total active aluminum (mg/kg) was extracted from 2.0 g of soil with 40 mL of 0.2 mol/L ammonium oxalate (pH 3.0) in the dark with shaking for 4 h, and quantified by ICP-OES (Agilent 5110 VDV, Agilent Technologies, USA). Exchangeable Al^3+^ (mg/kg) in the KCl extract was quantified by chromazurol S (CAS) spectrophotometry: 10 mL of extract was mixed with 2 mL of 0.1% CAS and 5 mL of acetate buffer (pH 5.5), reacted for 20 min, and measured at 625 nm (calibration range 0–5 μg/mL Al as AlCl_3_). Other operationally defined forms of active aluminum were extracted and determined following published methods [10]. All extractions were performed in triplicate (*n* = 3) with procedural blank corrections. Data were presented as mean ± standard deviation (SD).

Solid samples (RH, SD, and their biochars; acidic red soils before and after 180-day stabilization treatment) were dried at 60 °C, ground, and sieved to 100 mesh prior to compositional and structural characterization. The presence of carbon, hydrogen, and nitrogen was determined using an elemental analyzer, and K, Fe, Mg, Mn, Zn, Mo and other elements were quantified by ICP–OES following acid digestion [19]. Crystalline and amorphous mineral phases were characterized by X-ray diffraction (XRD) using Cu Kα radiation (λ = 1.5406 Å; 40 kV, 40 mA; 2θ = 5–80°, step size 0.02°) [19]. Specific surface area and pore structure data were obtained from N_2_ adsorption–desorption isotherms (BET/BJH; Micromeritics ASAP 2460, Micromeritics Instrument Corporation, Norcross, GA, USA) after vacuum degassing at 200 °C for ≥12 h. Micro-morphology was examined by field-emission scanning electron microscopy (SEM). Surface elemental valence states and chemical environments were analyzed by X-ray photoelectron spectroscopy (XPS) with peak fitting, with particular attention to C 1 s deconvolution [20]. Surface functional groups were characterized by Fourier transform infrared spectroscopy (FTIR; KBr pellets, 4000–400 cm^−1^) and nuclear magnetic resonance spectroscopy (NMR; Bruker, 500 MHz, Bruker Corporation, Karlsruhe, Germany).

**Microbiological and molecular analysis.** At the end of the batch experiment, each treatment (RH, RB, SD, and SB) was rapidly cryopreserved in liquid nitrogen. To analyze microbes attached to biochar and the bulk soil microbiome, samples were gently sonicated in sterile phosphate-buffered saline (PBS, 0.05% Tween-20) to desorb attached cells, then combined with soil for total genomic DNA extraction using the PowerSoil Pro kit (QIAGEN N.V., Venlo, The Netherlands); extraction blanks were included as negative controls. Extracted DNA was quantified, purity was assessed (A260/280 ≈ 1.8–2.0), and integrity was verified by 1% agarose gel electrophoresis [21]. The bacterial 16S rRNA V3–V4 region was amplified with universal primers 515F and 907R; PCRs were performed in triplicate and pooled. The thermocycling conditions were as follows: 95 °C for 3 min; 30 cycles of 95 °C for 30 s, 55 °C for 30 s, and 72 °C for 45 s; final extension at 72 °C for 5 min [22]. Amplicons were purified with AMPure XP beads, adapters were ligated to construct libraries, and paired-end sequencing (2 × 300 bp) was conducted on an Illumina MiSeq platform. Raw reads were quality filtered and adapter-trimmed with fastp, denoised to amplicon sequence variants (ASVs) using the QIIME 2–DADA2 pipeline (Illumina, lnc., San Diego, CA, USA), and taxonomically assigned against the SILVA 138 database.

## 3. Results and Discussion

### 3.1. Physicochemical Properties of Biomass and Biochar

**Figure 1:**
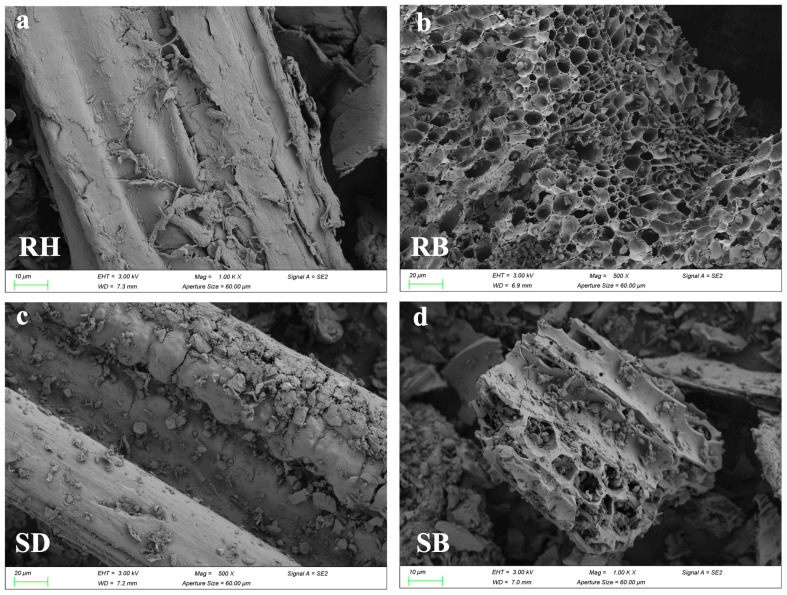

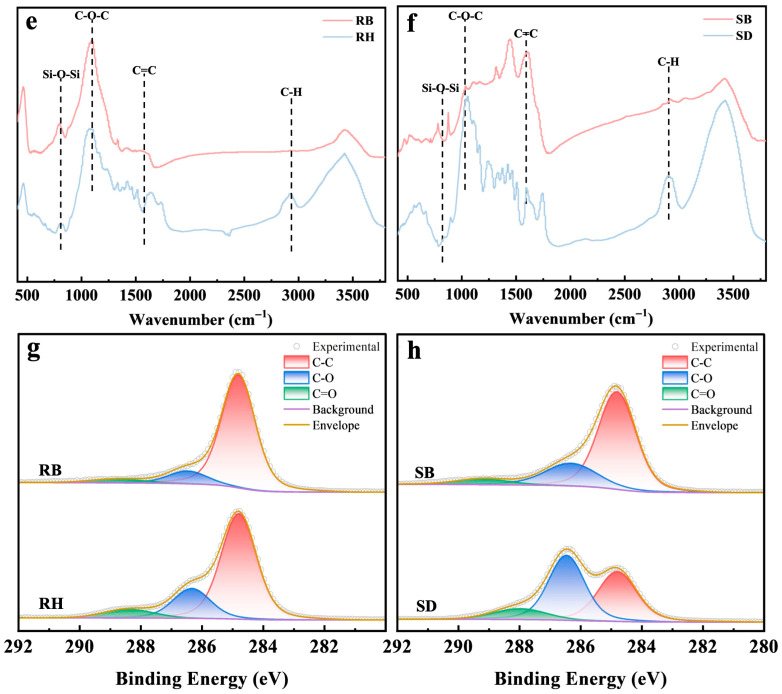
Surface morphology and chemical functionality of raw biomasses and their biochars produced at 550 °C: (**a**–**d**) SEM micrographs, (**e**,**f**) FTIR spectra highlighting functional group, and (**g**,**h**) high-resolution C 1 s XPS profiles revealing carbon speciation. Rice husk (RH), sawdust (SD), rice husk biochar (RB), and sawdust biochar (SB).

Figure 1 systematically revealed the multiscale structural evolution of RH and SD after pyrolysis. SEM images (Figure 1a,c) showed that the raw RH and SD surfaces were relatively dense and smooth. After pyrolysis at 550 °C, RB developed a honeycomb-like porous framework (Figure 1b), and its S_BET_ increased to 64.8 m^2^/g (Table S1). By contrast, SB exhibited micropores and fissures (Figure 1d), but its surface area increased by only about 55.1 m^2^/g. FTIR spectra (Figure 1e,f) showed pronounced aliphatic C–H stretching bands near 2920 cm^−1^ in the raw RH and SD, but the band intensities in RB and SB decreased markedly in this region, confirming extensive decomposition of aliphatic components [23]. Meanwhile, the aromatic C=C skeletal vibration near 1600 cm^−1^ was substantially enhanced in RB and SB [24], whereas the band associated with oxygen-containing groups (C–O–C) at ~1100 cm^−1^ decreased by more than 50%, indicating that pyrolysis promoted deoxygenation and dehydration, forming more stable aromatic carbon structures. Notably, RB exhibited a characteristic Si–O–Si band at ≈800 cm^−1^ [25], consistent with the inherently high ash content of rice husk, whereas this band was absent in SB, underscoring the pronounced influence of feedstock on biochar surface chemistry. Mechanistically, the spectral evolution indicated that the cleavage of aliphatic moieties together with enhanced aromatization improved biochar structural stability. XPS C 1s peak deconvolution (Figure 1g,h) and ^13^C NMR spectra (Figure S1) further confirmed a substantial increase in C–C relative to C–O/C=O functionalities [26,27], reflecting a sustained deoxygenative condensation process.

Elemental and structural characterization of the raw biomasses and their biochars (Table 1 and Table S1) indicated that pyrolysis markedly altered material properties, which were critical for environmental applications, particularly the stabilization of active aluminum in acidic red soils. Pyrolysis promoted ash formation, and RB exhibited a higher ash content than SB, consistent with the greater inorganic mineral content of RH [28]. The atomic H/C ratio, a key proxy for aromaticity, decreased substantially upon pyrolysis from 127.3 for RH to 54.6 for RB, and from 151.7 for SD to 88.5 for SB, indicating pronounced dehydration, decarboxylation, and condensation to aromatic structures during pyrolysis [29]. The predominance of amorphous Fe(III) oxides and water-soluble K^+^ in biochar might synergistically enhance aluminum stabilization, the former through covalent bonding with active aluminum in acidic red soil, the latter via pH-driven precipitation that concurrently enriched functional microbes for long-term immobilization. Meanwhile, the S_BET_ of both biochars increased rapidly with pyrolysis, reflecting the volatilization of organics and extensive development of micro- and mesopores [30]. The pronounced increase in S_BET_, together with enhanced aromaticity, suggested that, relative to the raw biomasses, RB and SB were more likely to stabilize active aluminum in acidic red soils via mechanisms such as pore entrapment and acting as microbial carriers.

### 3.2. The Stabilization Performance of Biomass and Biochar

Over a 180-day experiment, the 3% RB treatment significantly decreased the total active aluminum concentration from an initial 625 mg/kg to 428 mg/kg by day 180, yielding a stabilization efficiency of 22.1 ± 1.1%, which outperformed the RH treatment (16.1 ± 0.5%). This RB stabilization efficiency was consistent with a previous study of 26.74% with 2% biochar [10]. Similarly, for exchangeable Al^3+^, RB exhibited rapid stabilization by day 30, decreasing from 173 mg/kg initially to 104 mg/kg, and maintained a stable downward trend throughout the experiment. In comparison, the SB treatment also had a marked effect, but its stabilization efficiencies for total active aluminum and exchangeable Al^3+^ were approximately 5.1% and 8.3% lower than those of RB, respectively. Notably, the uncarbonized RH and SD showed limited capacity to stabilize total active aluminum and exchangeable Al^3+^ and even induced slight release during the first 110 days, likely due to transient pH depression caused by rapid mineralization of organic matter [31]. Prior studies further indicated strong co-regulation between biochar inputs and the soil microbiome. For example, biochar enriched root endophytic microbial populations such as *Nakamurella*, *Aureimonas*, *Luteimonas*, and *Sphingomonas*, whose metabolic products promoted complexation of Al^3+^ with organic ligands, thereby diminishing its activity [32,33]. Overall, the dominance of RB might stem from dual pH–element synergy [34]. The higher pH (8.2 ± 0.3) of RB triggered rapid Al(OH)_3_ nucleation, while Fe(III) surfaces enabled covalent Al^3+^ binding, together providing a neutral micro-domain niche for enriched functional microorganisms for sustained Al^3+^ immobilization.

**Figure 2:**
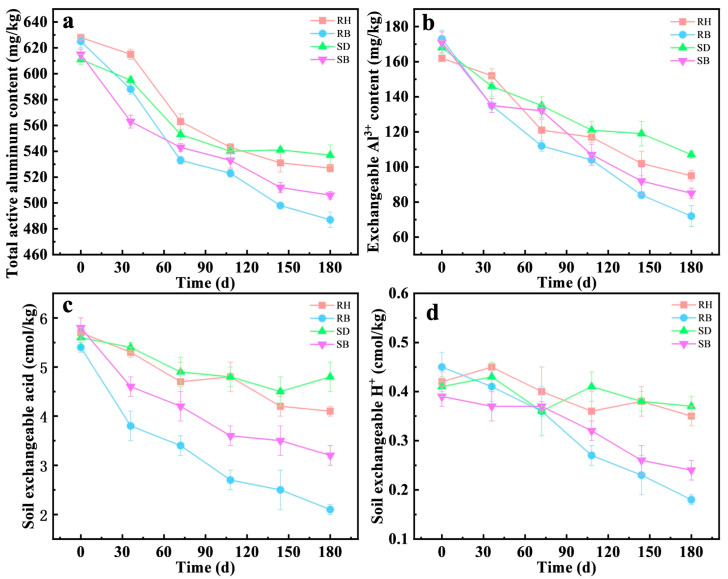
Temporal changes in (**a**) total active aluminum, (**b**) exchangeable Al^3+^, (**c**) soil exchangeable acid, and (**d**) exchangeable H^+^ in acidic red soils amended with 3% RH, SD, RB, or SB over 180 days (mean ± SD, *n* = 3). Rice husk (RH), sawdust (SD), rice husk biochar (RB), and sawdust biochar (SB).

Under the RB treatment, soil exchangeable acid decreased from an initial 5.4 cmol/kg to 2.1 cmol/kg by day 180, a decrease of 42.6%. The exchangeable H^+^ declined from 0.45 to 0.18 cmol/kg, corresponding to a 60.0% decrease. By contrast, the SB treatment lowered exchangeable acid to 3.2 cmol/kg, but decreased exchangeable H^+^ by only 38.5% (to 0.24 cmol/kg). In parallel, the higher ash content and specific surface area of RB (Table 1 and Table S1) conferred greater proton-neutralization capacity and ion-exchange capacity [35,36]. These results not only confirmed that RB achieved a sustained increase in soil pH via the combined effects of alkaline ash and a porous structure, but also suggested functional coupling with indigenous microorganisms. The persistent decline in exchangeable acidity occurred concomitantly with deep stabilization of active aluminum, implying that biochar-created microenvironments might have promoted the proliferation of alkalinity-generating or other relevant functional guilds, thereby indirectly mediating the precipitation of active aluminum, see Figure 2.

### 3.3. Key Factors of Biomass and Biochar Governing the Soil Active Aluminum

**Figure 3:**
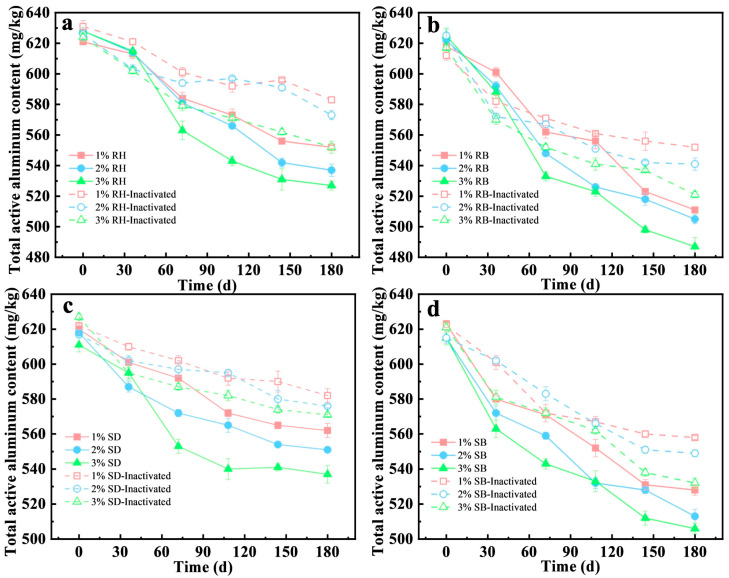
Influence of amendments of raw biomasses and their biochars under different addition amounts and microbial inactivation on the stabilization behavior of total vanadium from acid red soil (mean ± SD, *n* = 3): (**a**) rice husk (RH), (**b**) rice husk biochar (RB), (**c**) sawdust (SD), (**d**) sawdust biochar (SB).

The stabilization capacity for active aluminum in acidic red soil at 1%, 2%, and 3% (*w*/*w*) amendment rates (Figure 3a,b) was systematically evaluated. The results showed that biochar amendments exhibited pronounced aluminum stabilization with a clear dose dependence. At 3% dose, RB decreased the active aluminum concentration in soil from 625 mg/kg to 487 mg/kg, corresponding to a stabilization efficiency of 22.1%, which outperformed the 2% and 1% RB doses (18.8% and 13.7%, respectively). Environmentally, this strategy valorizes agricultural wastes, reducing landfill burden. However, 1% RB application was reportedly cost-effective for smallholder farms, mitigating Al^3+^ toxicity to improve crop root growth and nutrient uptake [37].

In contrast, raw biomass amendments showed limited efficacy. For example, RH at 3% achieved only 16.1% stabilization, approximately 5% points lower than RB. Notably, at identical dosing levels, the aluminum stabilization efficiency of RB was 1.3–1.6 times that of RH. Prior studies indicated that pyrolysis increased specific surface area and micropore volume [38], surface alkalinity, and the effective exposure of stabilization-relevant functional groups [39], thereby strengthening the chemical complexation and surface adsorption of active stabilization.

The stabilization efficiency was significantly decreased in the inactivated treatments (Figure 3a–d), underscoring the critical contribution of microbial processes to aluminum stabilization. For example, under the 3% RB treatment, the stabilization efficiency in the inactivated treatment was 15.6%, only 74% of that in the live treatment, indicating that microbial activity enhanced the immobilization of active aluminum. Similarly, for the RH and SD amendments at 3% dosage, the difference in stabilization efficiency between the live and inactivated groups was 5.7–6.0%. This effect was likely attributable to functional microorganisms elevating local microenvironmental pH to promote Al(OH)_3_ precipitation [40], and producing EPS that facilitated the formation of stable organo-metal complexes [41,42].

### 3.4. Properties Evolution of Biomass and Biochar

In the acidic red soil, additions of RH, SD, and their pyrolyzed biochars (RB and SB) significantly modulated the chemical speciation of active aluminum (Figure S2). Following the application of the raw biomasses (RH and SD), humic acid-bound aluminum (Al–HA) increased to 23% and 24%, respectively, suggesting that inputs of fresh organic matter may have released low-molecular-weight organic acids that promoted dissolution of mineral-phase aluminum. In contrast, the application of RB and SB reduced Al–HA to 18% and 17%, with decreases of 21% and 29%, while the proportion of Al(OH)_3_ increased to 64% and 58%, indicating that biochar promoted the transformation of active aluminum toward more stabilized forms [43].

**Figure 4:**
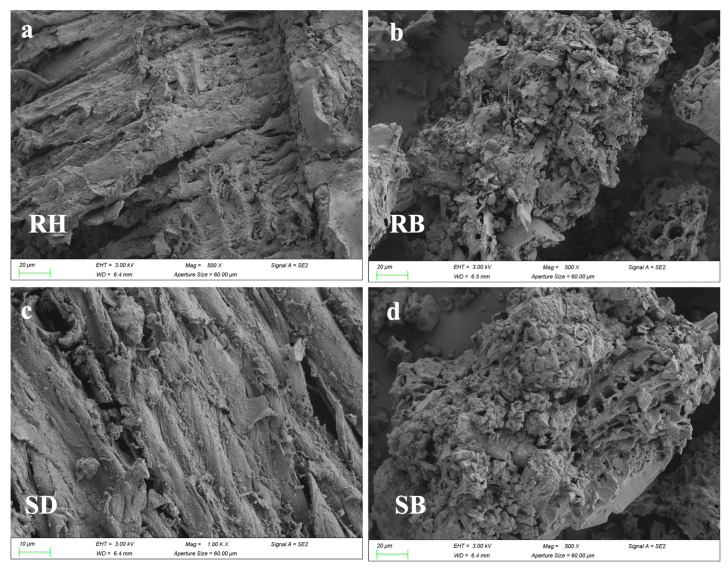

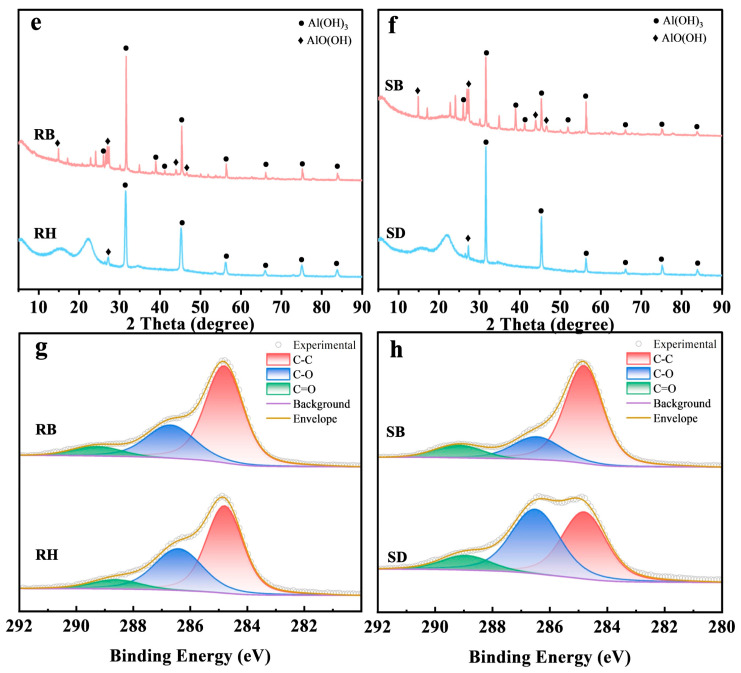
The characterization of the surfaces of raw biomasses and their biochars after aluminum stabilization: (**a**–**d**) SEM observations showing morphological alterations; (**e**,**f**) XRD spectra and (**g**,**h**) C 1s XPS profiles. Rice husk (RH), sawdust (SD), rice husk biochar (RB), and sawdust biochar (SB).

SEM images (Figure 4a–d) showed that after the 180-day experiment, microbial colonization density on RB and SB surfaces was higher than on RH and SD, and dense extracellular precipitate–biochar composites had formed. This indicated that high-temperature pyrolysis had enhanced surface roughness and microporosity, which favored microbial colonization. XRD patterns (Figure 4e,f) exhibited new reflections at 2θ = 38.4°, 41.8°, and 44.3°, assignable to Al(OH)_3_ and AlO(OH) precipitates [44]. Notably, in RB, the Al(OH)_3_ peak at 2θ = 31.5° displayed higher crystallinity with a sharper, more intense peak and a smaller full width at half maximum (FWHM), implying greater lattice order and larger effective crystallite sizes than in the other treatments. Together with the micro-morphological and surface-chemical evidence, these observations indicated that the porous carbon framework of RB provided ecological niches for indigenous Al-tolerant bacteria and likely induced microbially mediated local pH elevation, thereby promoting the transformation of active aluminum into low-solubility hydroxides.

As evidenced by the C 1s XPS spectra (Figure 4g,h), microbial colonization significantly enriched oxygenated functional groups on both raw biomasses and biochars. For rice husk (RH) and sawdust (SD), the carboxyl (–COOH) component at 289.0–289.5 eV intensified (Figure 4g), demonstrating microbially driven edge-site oxidation and surface carboxylation [45]. Concurrently, rice husk biochar (RB) and sawdust biochar (SB) exhibited enhanced carbonyl/ester (C=O) contributions at ~286.2 eV (Figure 4h), indicative of microbial oxidation and the structural rearrangement of residual aliphatic chains toward higher-polarity functionalities [46]. Collectively, these results reveal a microbial-mediated transition from “aromatic carbon-dominated” to “oxygenated functional group-enriched” interfaces on colonized biochars. This transformation constructs carboxyl/carbonyl coordination sites along aromatic edges, thereby markedly enhancing the selective adsorption of active aluminum species through ligand exchange and inner-sphere complexation (e.g., bidentate/bridging carboxylate configurations).

### 3.5. Dynamics and Stabilization Mechanisms of Microbial Community in Response to Aluminum Stress and Biochar Amendments

**Figure 5:**
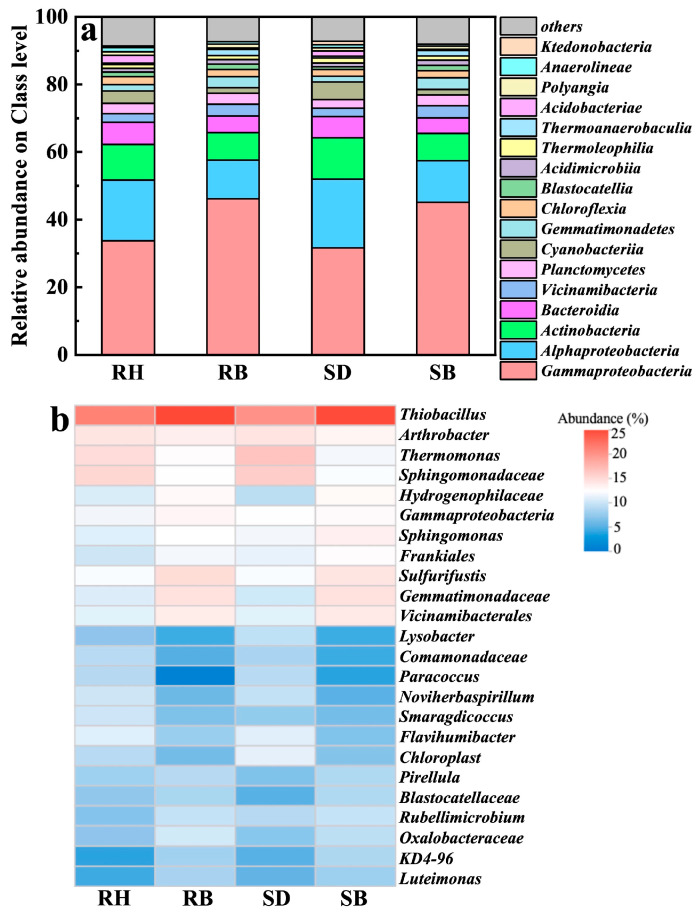
Soil microbial community structure after amendments with raw biomasses and their biochars: (**a**) relative abundance at the phylum level and (**b**) genus composition. Rice husk (RH), sawdust (SD), rice husk biochar (RB), sawdust biochar (SB).

High-throughput 16S rRNA gene sequencing revealed that four dominant bacterial classes, i.e., *Gammaproteobacteria*, *Alphaproteobacteria*, *Actinobacteria*, and *Bacteroidia*, collectively accounted for over 70% across all treatments (Figure 5a). Notably, the biochar-amended soils (especially RB and SB) exhibited elevated relative abundances of *Gammaproteobacteria*, which were 1.4- and 1.5-times those in the RH and SD groups, respectively. This clade encompassed numerous metal-tolerant and organotrophic genera that likely promoted Al^3+^ complexation and passivation via EPS secretion and/or microenvironmental pH modulation [47]. Concurrently, *Alphaproteobacteria*, taxa typically adapted to neutral soils, were also enriched in RB and SB, indicating that biochar increased soil pH, alleviated acid stress, and thereby expanded their available ecological niches [48].

At the genus level (Figure 5b), *Thiobacillus*, *Arthrobacter*, and *Thermomonas* were significantly enriched in the acidic red soils amended with RB and SB, with relative abundances of 24.5–25.0%, 2.3–2.5%, and 1.7–2.0%, respectively, and were especially pronounced under RB. *Thiobacillus* is a well-documented chemolithoautotrophic denitrifier to reduce nitrate to N_2_ under anoxic conditions [49,50], and it has been shown to maintain high metabolic activity under heavy-metal stress [51]. In this study, its marked enrichment suggested that biochar-induced reducing microdomains promoted niche expansion and may have indirectly regulated active aluminum speciation, by modulating microscale pH and redox potential to suppress Al^3+^ dissolution kinetics [52].

*Arthrobacter*, a canonical metal-tolerant genus widespread in contaminated soils, possesses broad carbon-use capacity and can sequester multiple cations (e.g., Al^3+^, Cu^2+^) through extracellular complexation or intracellular compartmentalization [53,54,55]. Its elevated abundance in the biochar treatments indicated that oxygenated surface functionalities and porous structures provided both an adsorptive barrier and microhabitats, thereby enhancing resistance to aluminum toxicity and facilitating colonization. *Thermomonas*, originally isolated from thermophilic compost and recently linked to nitrate reduction, coupled with organic matter degradation, particularly in environments rich in aromatic compounds [56,57]. This pattern suggested that increased biochar aromatization may have released intermediate metabolites favorable for its metabolism, forming a synergistic carbon scaffold–functional guild feedback. Moreover, these functional genera were more abundant under RB than SB. Coupled with RB’s higher ash content, we inferred that RB not only facilitated surface precipitation to immobilize free Al^3+^, but, owing to its alkalinity, high specific surface area, and porosity, also provided neutral microdomain niche for denitrifiers and stable mineral–interfacial attachment sites for aluminum-tolerant microbes. On the other hand, denitrifier colonization might elevate micro-environmental pH via alkaligenic metabolism, driving Al^3+^ precipitation as low-toxicity Al(OH)_3_ at biochar–solution interfaces, collectively eliminating root phytotoxicity, restoring rhizosphere nutrient uptake, and promoting denitrifier-dominated microbiome assembly to elevate crop stress resilience in acidic soils.

**Figure 6:**
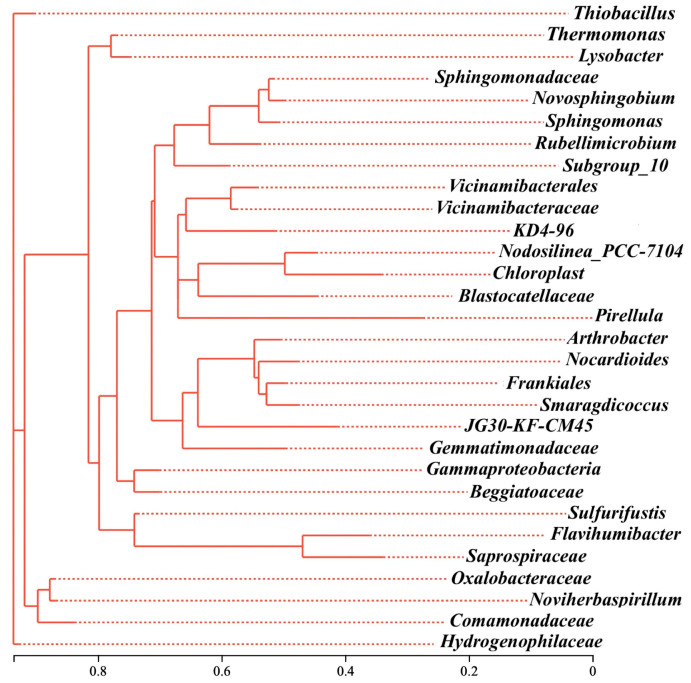
The evolutionary tree of main denitrifiers at genus level.

The phylogenetic analysis further showed that those three *genera* were distributed across distinct evolutionary lineages within the *Proteobacteria*, and in the shared microbial community of RB, SD, and SB, as shown in Figure S3. *Thiobacillus* was affiliated with *Hydrogenophilaceae*, *Thermomonas* with *Micrococcaceae*, and *Arthrobacter* with *Xanthomonadaceae* (Figure 6). The substantial genetic distances between them indicated that biochar-driven microbiome restructuring was not confined to the expansion of a single lineage but rather reflected a functional selection process that spanned phylogenetic boundaries. This cross-domain/cross-class remodeling mechanism suggested that the role of biochar extended beyond merely altering carbon-source availability. It more profoundly reshaped energy-flow pathways and ecological interaction networks in soil, particularly under extreme conditions (e.g., acidic red soils) by synergistically buffering pH, immobilizing toxic metal ions, and prolonging organic carbon residence time to modulate the colonization dynamics of key functional taxa.

### 3.6. Potential Mechanisms of Biochar Coupling Denitrifiers to Stabilize Active Aluminum in Acidic Red Soils

In acidic red soils, nitrate and soil organic carbon often co-accumulate in the soil layer (0–30 cm) due to excessive fertilizer application [58]. As a high-potential electron acceptor, nitrate could enhance denitrification flux within the anoxic microdomains of aggregates and stimulate the metabolic activity of denitrifying microbes using soil organic carbon as a carbon source. As depicted in Figure 6, RB and SB, by virtue of their intrinsic alkalinity (pH > 8) and highly developed porosity (S_BET_ up to 55.1–64.8 m^2^/g), created stable, locally neutral microdomains within the strongly acidic matrix [59]. They effectively buffered pH fluctuations in the surrounding environment and provided suitable interfacial habitats for functional microorganisms such as *Thiobacillus*, *Arthrobacter*, and *Thermomonas* that possessed organic oxidation capacity and denitrification potential.

**Figure 7:**
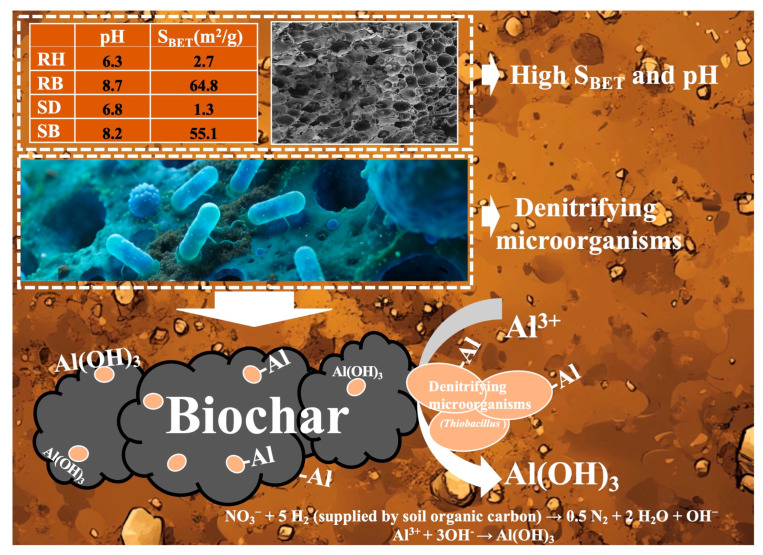
Schematic of biochar–denitrifier coupled stabilization of active aluminum in acidic red soil. Rice husk (RH), sawdust (SD), rice husk biochar (RB), sawdust biochar (SB), and specific surface area (S_BET_).

Upon colonizing biochar surfaces (Figure 7), these microbes further elevated microenvironmental pH through alkalinity-generating metabolism [NO_3_^−^ + 5 H_2_ (supplied by soil organic carbon) → 0.5 N_2_ + 2 H_2_O + OH^−^] [40,42], thereby driving the transformation of dissolved Al^3+^ into low-toxicity Al(OH)_3_, which precipitated at the char–solution interface. Meanwhile, the long-term attachment of microorganisms markedly increased the density of polar moieties such as carboxyl (–COOH) and hydroxyl (–OH), thus transforming the biochar from a largely nonspecific sorbent into a biohybrid material with high selective coordination capacity for active aluminum.

## 4. Conclusions

This study demonstrates that rice husk biochar (RB) is the optimal amendment for stabilizing active aluminum in acidic red soil, achieving 22.1 ± 1.1% total active Al reduction and 58.4 ± 3.5% exchangeable Al^3+^ reduction via its high ash content, porosity, and pH. Biochar-created neutral microdomains enriched denitrifiers (*Thiobacillus*, *Arthrobacter*, *Thermomonas*) that elevate pH and enhance oxygen-containing functional groups, driving Al(OH)_3_ precipitation and ligand exchange. Valorizing agricultural waste, this biochar–microbe strategy offers a scalable, climate-friendly solution for long-term Al toxicity mitigation, with future research focusing on field persistence and soil–plant system dynamics.

## Figures and Tables

**Table 1:** Elemental and S_BET_ analysis of the biomass and its derived biochars—rice husk (RH), sawdust (SD), rice husk biochar (RB), sawdust biochar (SB)—and specific surface area (S_BET_).

	Ash Content (%)	C (%)	O (%)	H (%)	N (%)	S (%)	C/N	H/C	O/C	pH	S_BET_(m^2^/g)
RH	/	38.2	55.8	5.6	0.3	0.12	148.56	1.76	1.10	6.3	2.7
RB	60.2	65.5	10.7	2.3	1.2	0.11	63.68	0.42	0.12	8.7	64.8
SD	/	45.5	48.7	5.5	0.3	0.05	176.94	1.45	0.80	6.8	1.3
SB	18.2	70.8	16.0	2.3	0.8	0.07	103.25	0.39	0.17	8.2	55.1

## Data Availability

The original contributions presented in this study are included in the article/Supplementary Material. Further inquiries can be directed to the corresponding authors.

## Supplementary Materials

The following supporting information can be downloaded at https://www.mdpi.com/article/10.3390/toxics14020157/s1: Table S1: Elemental content of biochar; Table S2: Stabilization efficiencies of total active aluminum at different doses of RH and RB in acidic red soil; Table S3: Stabilization efficiencies of total active aluminum at different doses of SD and SB in red acid soil; Figure S1: NMR ^13^C patterns of rice husk biochar (RB); Figure S2: The percentage of active aluminum in the red acid soil amended with rice husk (RH), sawdust (SD), rice husk biochar (RB), and sawdust biochar (SB); Figure S3: Venn diagrams of the shared and unique number of identified genus in red acid soil amended with rice husk (RH), sawdust (SD), rice husk biochar (RB), and sawdust biochar (SB).
