# Supplementary material for: Stabilizing Active Aluminum (Al3+) in Acidic Soils via Biochar-Induced Microbial Niches: Focusing on Denitrifier-Mediated Mechanisms, Efficiency, and Environmental Outcomes
Source: Toxics. 2026 Feb 6;14(2):157. doi: 10.3390/toxics14020157 (PMC12944886; doi:10.3390/toxics14020157)
Supplement: Supplementary file 1 [file toxics-14-00157-s001.zip › toxics-4095472-supplementary.pdf]

Supplementary Information

This supporting information contains:

number of pages: 6

number of tables: 3

number of figures: 3

Table S1. Elemental content of biochar

Biochar	Elemental content（**mg/g**）					
	**Mg**	**K**	**Fe**	**Mn**	**Zn**	**Mo**
**RB**	0.623±0.031	1.783±0.824	1.901±0.062	0.017±0.010	0.002±0.001	0.009±0.003
**SB**	0.241±0.03	1.421±1.035	0.152±0.005	0.015±0.013	0.002±0.001	0.002±0.001

Table S2. Stabilization efficiencies of total active aluminum at different dose of RH and RB in red acid soil.

**Material**	**RH**			**RB**		
**Dosage**	**1%**	**2%**	**3%**	**1%**	**2%**	**3%**
**Stabilization efficiency (%)**	11.1	14.5	16.1	17.3	18.8	22.1
**SD (%)**	0.2	0.6	0.5	0.2	0.5	1.1

Table S3. Stabilization efficiencies of total active aluminum at different dose of SD and SB in red acid soil.

**Material**	**SD**			**SB**		
**Dosage**	**1%**	**2%**	**3%**	**1%**	**2%**	**3%**
**Stabilization efficiency (%)**	9.4	10.8	12.1	15.2	16.6	17.8
**SD (%)**	0.6	0.2	0.8	0.5	0.7	0.5

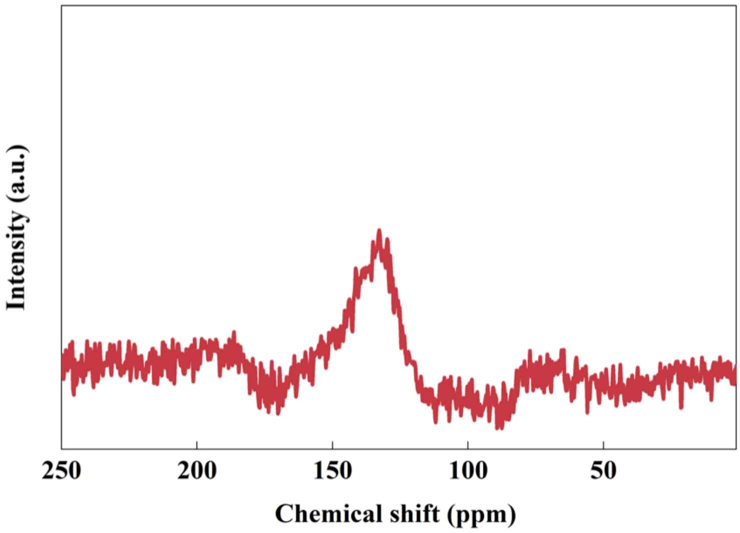

Fig. S1. NMR ^{13}C patterns of rice husk biochar (RB)

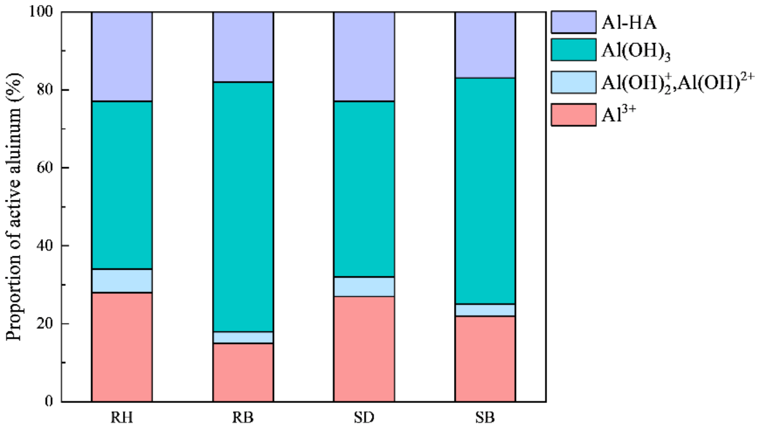

Fig. S2. The percentage of active aluminum in the red acid soil amended with Rice husk (RH), Sawdust (SD), Rice husk biochar (RB) and Sawdust biochar (SB).

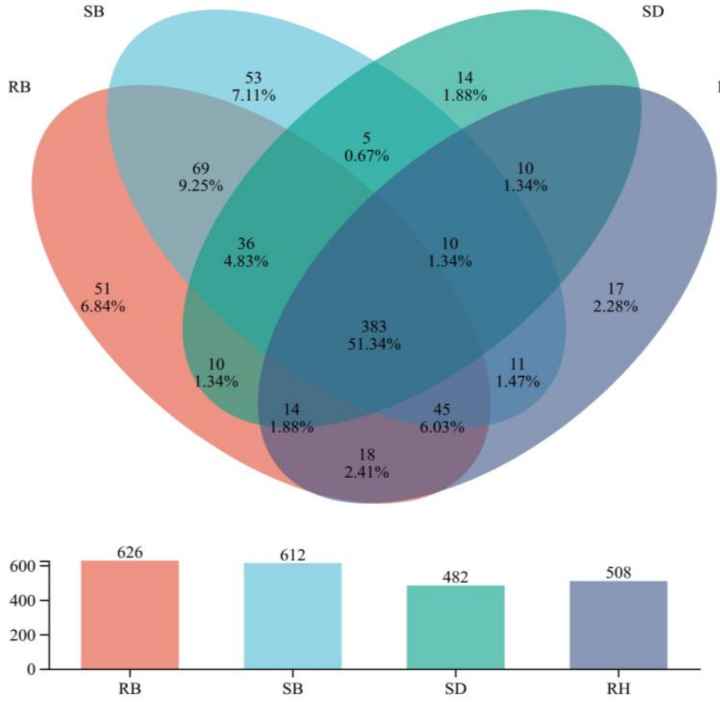

Fig. S3. Venn diagrams of the shared and unique number of identified genus in red acid soil amended with Rice husk (RH), Sawdust (SD), Rice husk biochar (RB) and Sawdust biochar (SB).
